# Information distribution patterns in naturalistic dialogue differ across languages

**DOI:** 10.3758/s13423-024-02452-0

**Published:** 2024-01-24

**Authors:** James P. Trujillo, Judith Holler

**Affiliations:** 1 Donders Institute for Brain, Cognition, and Behaviour, Nijmegen, The Netherlands; 2 Max Planck Institute for Psycholinguistics, Nijmegen, The Netherlands

**Keywords:** Cross-linguistic, Surprisal, Information distribution, Conversation, Turn-taking, Utterance planning

## Abstract

The natural ecology of language is conversation, with individuals taking turns speaking to communicate in a back-and-forth fashion. Language in this context involves strings of words that a listener must process while simultaneously planning their own next utterance. It would thus be highly advantageous if language users distributed information within an utterance in a way that may facilitate this processing–planning dynamic. While some studies have investigated how information is distributed at the level of single words or clauses, or in written language, little is known about how information is distributed within spoken utterances produced during naturalistic conversation. It also is not known how information distribution patterns of spoken utterances may differ across languages. We used a set of matched corpora (CallHome) containing 898 telephone conversations conducted in six different languages (Arabic, English, German, Japanese, Mandarin, and Spanish), analyzing more than 58,000 utterances, to assess whether there is evidence of distinct patterns of information distributions at the utterance level, and whether these patterns are similar or differed across the languages. We found that English, Spanish, and Mandarin typically show a back-loaded distribution, with higher information (i.e., surprisal) in the last half of utterances compared with the first half, while Arabic, German, and Japanese showed front-loaded distributions, with higher information in the first half compared with the last half. Additional analyses suggest that these patterns may be related to word order and rate of noun and verb usage. We additionally found that back-loaded languages have longer turn transition times (i.e., time between speaker turns).

## Introduction

In human interaction we use language to engage with one another. Typically, the goal of language is to influence another person’s mental state or future actions in some way by conveying information. One important constraint in language use is that words are produced one at a time. Listeners then process these “pieces” of information (i.e., words) as they are produced, allowing the listener to continuously update their interpretation of the utterance based on this incoming information and to plan a fitting next utterance. An important question in understanding language use and processing is how information is organized during an utterance, which would facilitate future investigations into how this organization may impact on turn-taking during naturalistic conversation (Levinson, 2016; Stivers et al., 2009).

One influential theory of information organization is the Uniform Information Density (UID) hypothesis (Jaeger, 2006; Jaeger & Levy, 2006). The UID hypothesis was initially used to explain variation in word length, with high-frequency words being typically shorter than low-frequency words. This leads to speakers conveying a smooth distribution of information over unit time, at the word level (Frank & Jaeger, 2008), allowing listeners to keep up with the spoken information. In other words, less frequent high-information words are typically longer and thus require longer to process, which means that the transmitted information is conveyed over a longer period of time. Collins (2014) provided evidence that the UID hypothesis is a strong predictor of real-world language use in the context of English syntactic structure. Collins (2014) further suggests, beyond specific syntactic constructions, that the UID hypothesis should also hold at the level of information distribution across an utterance. This would be seen as a smooth transition between low- and high-informative words across the utterance (i.e., the informativity of a word will be similar to the words before and after it), rather than a random, jagged distribution of low- and high-informative words (i.e., a high-informative word followed by a very low-informative word, followed by a high-informative word; Collins, 2014). Such a smooth organization ensures that information is distributed in such a way that it is more robust to noise, due to information being spread evenly (i.e., random deletion is less likely to critically affect information transfer), and is also efficient in terms of transmission time due to a relatively constant rate of information transfer. Conversely, Meister and colleagues (2021) suggest that such utterance-level uniformity may not hold, or may differ, between languages. Importantly, however, the authors also note that little is known about information distribution at the utterance level.

While some studies have investigated information organization in language, most studies either focus on a single language or focus on written text rather than dialogue, where most language use takes place. One recent study has addressed the issue of utterance-level information distribution and how this may differ across languages. Klafka and Yurovsky (2021) performed an analysis of 234 languages, using text from Wikipedia articles, showing that there is a smooth distribution of information across sentences. Furthermore, the authors use an information curve, plotting surprisal values across sentences, to show that while some language families show a gradually increasing curve of information, others show a decreasing curve, and yet others show a mix (Klafka & Yurovsky, 2021). This shows that while information density is smooth, it is not entirely uniformly (i.e., flatly) distributed within an utterance. Instead, there seems to be a trend for information to be either front loaded (higher information density in the first half of a sentence) or back loaded (higher information density in the second half of a sentence). Such patterning of information distribution should be particularly important for language produced in dialogue, given that the processing of that language then takes place in the fast-paced back-and-forth of interaction, where whether information is front loaded versus back loaded may crucially matter when comprehending incoming information while also planning the responding turn at the same time. In fact, prior research showed that information processing during the second half of a turn in experimental settings was impacted by parallel next turn planning (Barthel, 2021; Bögels et al., 2018), which raises the possibility that similar constraints arise during natural conversations. While Klafka and Yurovsky (2021) show that English shows the same back-loaded information pattern whether in written or in spoken form, the spoken modality has not been investigated for other languages. Given the interactionally embedded nature of typical language use, it is especially important to investigate conversational language in order to understand how information distribution is organized in different languages (i.e., whether there are consistent patterns of front loading versus back loading). This will allow future research to experimentally test how this may impact the interactive partner’s processing and response planning.

The current study aims to assess information distribution across languages in conversational context. First, we employed a cross-linguistic assessment of telephone dialogues in six languages, collected as part of one larger corpus, to assess whether distinct information profiles are also observed in the spoken language. Making use of such a large dataset of comparable conversational corpora allowed us to perform a robust cross-linguistic analysis of conversation, rather than text. Our results therefore bring our understanding of information distribution closer to the natural ecology of language use: dialogue. Second, based on previous research, we will specifically test the hypothesis that information distribution is either front-loaded or back-loaded, depending on the language, providing a quantification of the different types of information curves that are visualized in Klafka and Yurovsky (2021). This quantification will be particularly relevant for our understanding of the mechanics of language in social interaction.

## Methods

### Corpus data and preparation

The data used in this study come from the CallHome corpus, which consists of unscripted conversations, conducted via telephone calls and lasting up to 30 minutes between pairs (i.e., dyads) of acquaintances, collected by the Linguistic Data Consortium (MacWhinney, 2007). CallHome consists of corpora from several languages. We utilized data from the Arabic, German, English, Japanese, Spanish, and Mandarin, as these corpora were openly available. All conversations were conducted between native speakers of the designated language. For these corpora, we utilized the per-utterance transcriptions, which covered a continuous stretch of 5 to 10 minutes of the total call. The number of dyads, number of utterances, and mean number of words per utterance can be found for each language in Table 1. Before performing any calculations, we first removed punctuation and non-lexeme annotations including laughter as well as filled pauses such as “uh,” “hm,” or “ah.” Importing and preparing the transcriptions was performed in Python (Version 3.7) using custom scripts.

**Table 1:** Corpus information per language

	Arabic	German	English	Japanese	Spanish	Mandarin
Dyads	200	120	178	120	140	140
Utterances in corpus** (***n***)**	33,120	35,105	37,000	40,482	32,203	40,458
Utterances after processing** (***n***)**	6,355	8,017	10,141	11,887	10,394	11,350
Mean words per utterance	4.455±3.54	4.810±4.23	5.596±4.97	5.744±5.43	5.912±4.67	5.839±5.47
Mean words per utterance after processing	9.440±1.29	10.213±1.69	11.404±2.49	12.525±3.76	11.321±2.19	12.746±4.31

### Word surprisal calculation

**Fig. 1:**
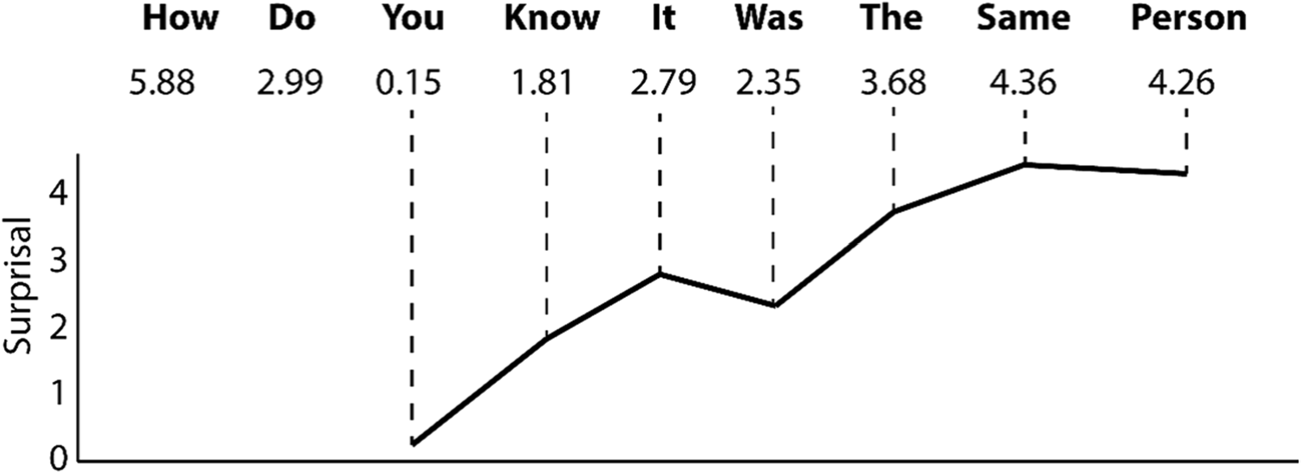
Illustration of an utterance taken from the English language corpus with corresponding surprisal values. Beneath the individual words and values, surprisal is plotted as an information curve with words represented along the *x*-axis and surprisal on the *y-*axis. Note that the surprisal of the first two words is not plotted on the information curve. This is because although surprisal values for the first two words are calculated, they are based on less context (i.e., fewer preceding words) than all subsequent words in an utterance. These first two words were therefore excluded from analysis, given that they are not complete trigrams

Calculations were done using in-house developed Python (Version 3.7; Van Rossum & Drake, 2009) scripts. We used the nltk python package (Bird et al., 2009) to train trigram (i.e., second order Markov) models of word co-occurrence on the transcriptions of each of the corpora. Trigram models take each utterance in the training corpus and count how many times a given word occurs after a given two-word sequence. For example, in English, the two-word sequence “Are you” could be followed by “going,” “done,” “ready.” By counting the number of times each possible third word occurs after a given sequence relative to the number of possible third words, we can calculate the probability of that third word occurring. The negative log of this probability is then calculated as the *surprisal* value associated with that word, and can be considered the unexpectedness of encountering that word given the context of the two preceding words (Hale, 2001; Levy, 2008). See Fig. 1 for an illustration of how surprisal values map onto words in an utterance.

After calculating the probabilities for all trigrams in a corpus, we created a subset of that corpus to exclude short utterances. Specifically, we excluded any utterances that contained fewer than eight lexical tokens, or that were less than 600 ms in duration. See Table 1 for an overview of how many utterances were included after this processing step. Next, we split our utterances into a first half and a last half. This was done based on a simple split on the number of words in the utterance (i.e., a 10-word utterance has five words in the first half and five in the last half). Mean surprisal values were then calculated separately for the first and the last halves. For the first half, we additionally removed the first two values, given that these were not proper trigrams (i.e., the first word of an utterance has no preceding context in this model, and the second word is effectively only a bigram). By excluding short utterances and removing the first two words from the mean surprisal calculations, we ensure that our results are not biased towards high mean surprisal values in the first half that could be due to these less-informed words, while simultaneously ensuring at least two complete, independent trigrams in each half (i.e., words 1–3 and 2–4 in the first half, and 5–7 and 6–8 in the last half).

### Analyses

All analyses were performed in RStudio (Version 1.1.463). In order to assess whether surprisal differed between the first and the last half of utterances, we built linear mixed-effects models using the lme4 package (Bates et al., 2015, p. 4). For each corpus, we first created a null model with surprisal as the dependent variable, number of words as a fixed effect, and dyad/speaker as a nested random intercept. Random slopes were not included, as this led to singular model fits. Using likelihood ratio tests of model comparison, we compared this model against the same model that also included utterance half (binary variable: first or last). This tests whether there is a difference between the first and last halves of the utterances, and specifically whether the first half shows higher or lower surprisal compared with the last half. In order to account for the number of tests being performed, we applied a Bonferroni correction and lowered our alpha threshold from 0.05 to 0.008. For each language corpus, we report the results of the model comparison, and the fixed-effect estimate taken from the model.

Finally, while our main question was whether each individual language showed a particular pattern of front or back loading, we were also interested in whether there were cross-linguistic differences in these patterns. We therefore also tested a model with surprisal difference (*surprisal last half − surprisal first half*) as the dependent variable, and language corpus as the main predictor of interest. This model, as well as the base model that it was compared against, also included number of words per utterance as a fixed effect, and a nested random effect of dyad/speaker. We then used emmeans to compute pairwise contrasts between each language, with Tukey’s adjustment of *p* values. As a complementary analysis, we also aimed to assess surprisal at the word level, with surprisal as dependent variable, utterance half and language corpus as fixed effects, and dyad/speaker as a nested random intercept. However, these models failed to converge, and thus are not reported further. We provide the scripts and data for these analyses alongside our main data and scripts (see subsection Data Availability).

## Results

For some languages, we found higher surprisal in the last half of the utterance, such as in English, model comparison: χ^2^(1) = 45.714, *p* < .001, fixed-effect estimate: 0.021, *t* = 6.765); Spanish, χ^2^(1) = 72.439, *p* < .001, fixed-effect estimate: 0.033, *t* = 8.518; and Mandarin, model comparison: χ^2^(1) = 950.23, *p* < .001, fixed-effect estimate: 0.233, *t* = 31.154.

**Fig. 2:**
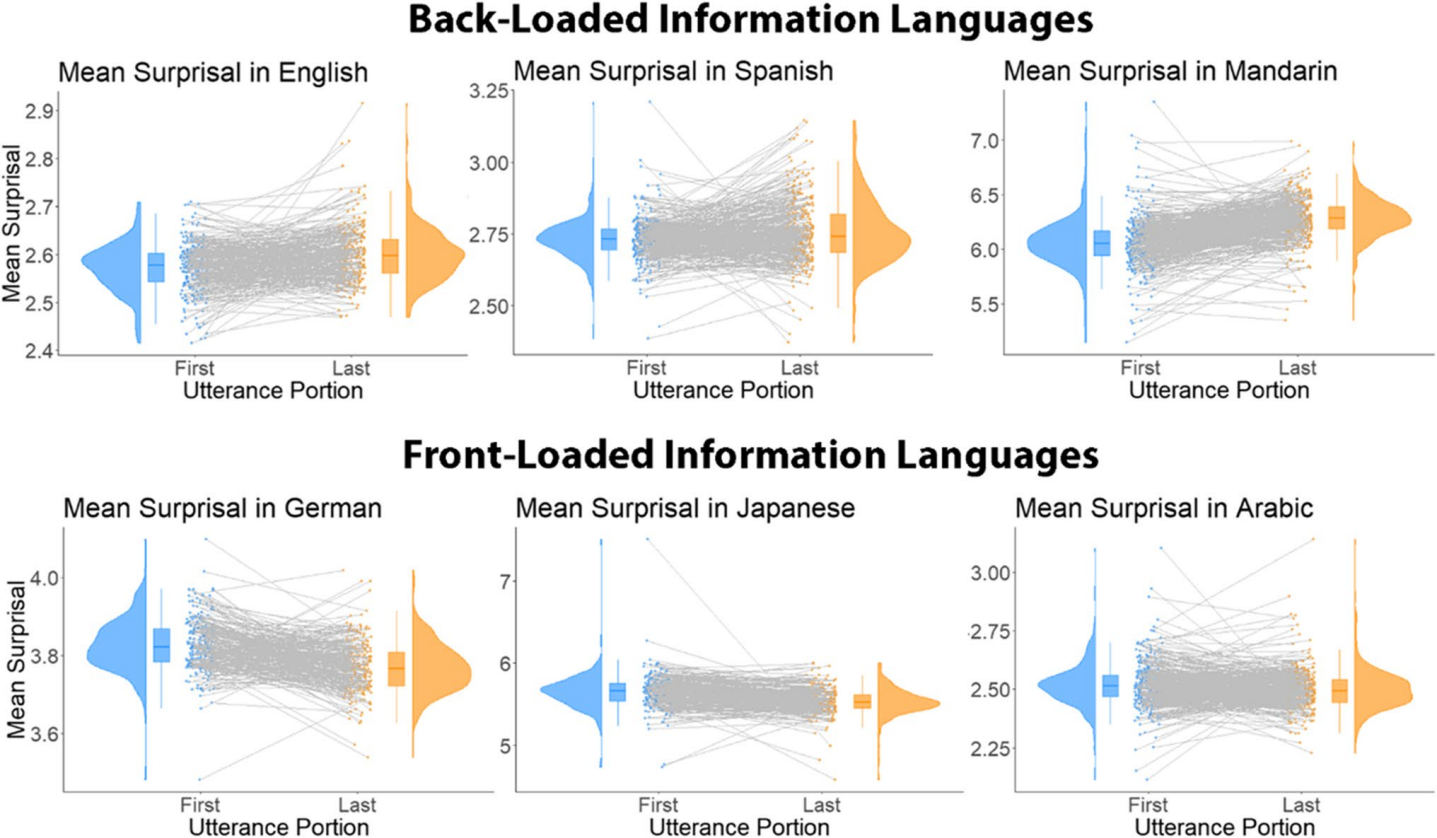
Overview of per-language differences between first and last utterance halves. The top row shows the back-loaded languages (i.e., those with higher surprisal in the Last half) and the bottom row shows the front-loaded languages (i.e., those with higher surprisal in the first half). For each graph, utterance half (first or last) is given on the *x*-axis, while surprisal is given on the *y*-axis. The boxes depict the median (center line) and 25% and 75% percentiles (upper and lower bounds of the box). Individual speaker means are given as black dots, connected by a line between First and Last half for each utterance

For other languages, we found higher surprisal in the first half, including German, model comparison: χ^2^(1) = 162.76, *p* < .001, fixed-effect estimate: −0.056, *t* = −12.790; Arabic, model comparison: χ^2^(1) = 26.766, *p* < .001, fixed-effect estimate: −0.021, *t* = −5.176; and Japanese, model comparison: χ^2^(1) = 226.460, *p* < .001, fixed-effect estimate: −0.115, *t* = −15.080. See Fig. 2 for an overview of these results.

**Fig. 3:**
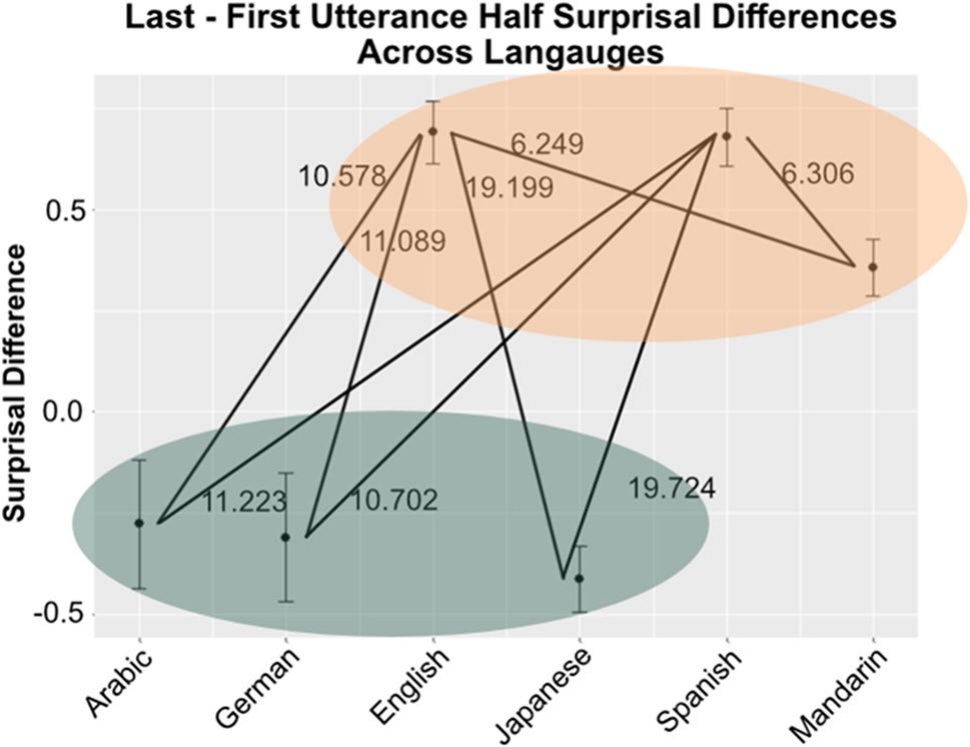
Cross-linguistic differences in between-utterance-half surprisal differences. The six languages are given along the *x*-axis, and the expected (marginal) surprisal difference (last half minus first half) is given on the *y*-axis. Lines indicate significant cross-linguistic contrasts, and the *z*-ratio values for each of these contrasts is given next to each line. Back-loaded languages are highlighted with a yellow ellipse, while front-loaded languages are highlighted with a green ellipse. Note that the two groups are clearly separated by, and show no overlap with, zero

Our test of the cross-linguistic model showed evidence that surprisal differences between first and second (utterance) half differed between languages, χ^2^(5) = 448.55, *p* < .001. Mean (centered) number of words was positively associated with surprisal differences (estimate = 0.004±0.0009, *t* = 4.422). Full parameter estimates for this model can be found in the Supplementary Material. Pairwise comparisons confirmed that the back-loaded languages (English, Spanish, and Mandarin) all differed from all of the front-loaded languages (German, Japanese, Arabic), with *p* values < .001. Within the back-loaded group, we also see that English and Spanish show a greater surprisal difference (i.e., more strongly back-loaded) than Mandarin (*p* < .001). We found no differences between languages within the front-loaded group. See Fig. 3 for an overview of these results.

### Post hoc analyses

#### Differences in rates and informativity of nouns and verbs

While the primary analyses suggest that, based on the information distribution measurements applied here, the six languages largely fall into two groupings (i.e., front and back loaded), an open question is what factors contribute to this difference in the distribution of information density. We therefore performed an additional set of analyses to determine if there are other measurable, systematic differences between these two groups. Our analyses focus on the contribution of word order, in the sense of distribution of particular grammatical units within an utterance. Specifically, we are using the term “word order” to refer to the relative placement of particular grammatical units within an utterance, such as whether nouns occur in the first or second half of an utterance. Focusing on the split between first and last half of the utterance is also in keeping with the broad split in utterance used for our other analyses. These analyses are based on work by Roberts and Levinson (2017), as well as Maurits (2012), who have proposed that word order, and in particular the placement of highly informative grammatical units such as nouns and verbs, would influence how information is organized and thus the processing of information by an addressee. However, word placement was not linked to information density patterns, or cross-cultural patterns of information distribution in these studies. The aim of these post hoc analyses was thus to assess (1) whether nouns and verbs occurred more frequently in the first half of front-loaded languages and the last half of back-loaded languages, and (2) whether nouns and verbs differed in their mean surprisal according to whether they were in the more information-dense half of an utterance (i.e., first half of front-loaded languages, or last half of back-loaded languages). These analyses were thus aimed to provide a theoretically motivated explanatory factor for why a particular utterance half shows higher information density than the other half. In particular, if nouns or verbs more frequently occur in the more information-dense half of an utterance, this could suggest that the syntax of these languages results in highly informative grammatical units occurring in different positions in the utterance depending on the language, which then leads to the information distribution patterns observed in our first analyses. Additionally, finding that the surprisal of nouns and verbs differ when in the more information-dense half of an utterance compared with the less information dense utterance half would provide some evidence that these languages differ not only in terms of the distribution of particular grammatical units within an utterance, but more generally in the way information is distributed, and thus how informative each word is. This analysis will therefore be informative for future studies investigating when in an utterance crucial information occurs, what types of grammatical units carry this information, and how this may impact turn-taking timing, or the processing of unfolding information (Maurits, 2012; Roberts & Levinson, 2017).

For the present analyses, rate was defined as the number of occurrences of a particular grammatical unit, divided by the total number of words in the utterance. In other words, if there were three verbs in an utterance half, and seven words total in that utterance half, then the mean surprisal of those three verbs was taken as the mean verb surprisal for that utterance half. Similarly, 3/7 (three verbs, seven words total) = 0.429, was taken as the verb rate for that utterance half. First/last half position and rate are then tied to their informativeness (i.e., surprisal), and how any such patterns of interaction between these factors differ according to the language grouping (i.e., front or back loaded).

We used the Python package Stanza (Qi et al., 2020) to perform automatic parts-of-speech tagging for all of the corpora we used. This provides us, for each word in each utterance, a universal part-of-speech tag, such as noun, verb, adverb, conjunction (henceforth “grammatical unit”). Note that this automatic tagging only provides a single, most-probable tag for each word, based on the lexical item and the grammatical units before and after a current word. Then, using the same surprisal values described above, we calculated, per utterance half, the mean surprisal of each grammatical unit, as well as the rate of occurrence per grammatical unit.

We then first created linear mixed models, with dyad and speaker as nested random effects, and either rate or surprisal as the dependent variable. In a step-wise fashion, we then tested whether the addition of grammatical unit (noun vs. verb), front/back grouping, and utterance half led to a significant increase in model fit. The three predictor variables were sum coded for these models. For the model with rate as dependent variable, we used the package glmmTMB (Brooks et al., 2023) and fit a beta distribution. We additionally included interaction terms between these fixed effects. This allowed us to test whether, for example, surprisal of a particular grammatical unit differs according to which front/back grouping it occurs in, and whether it differs according to where in the utterance it occurs (i.e., first or last half). This analysis provides an indication of how rate of occurrence and surprisal at the word level are associated with the utterance half and grammatical unit of the word.

For these analyses, we specifically predicted that nouns and verbs would occur more frequently in the utterance half that was found to be higher in overall information density. In other words, we expected noun and verb rate to be associated with an interaction between front versus back loading and utterance half. This would indicate that the differences in information distribution across languages may be partially due to the grammatical structure of the language. Our choice to model rate and surprisal as the dependent variables is therefore reflected in our predictions regarding interactions between front versus back loading and utterance half.

**Fig. 4:**
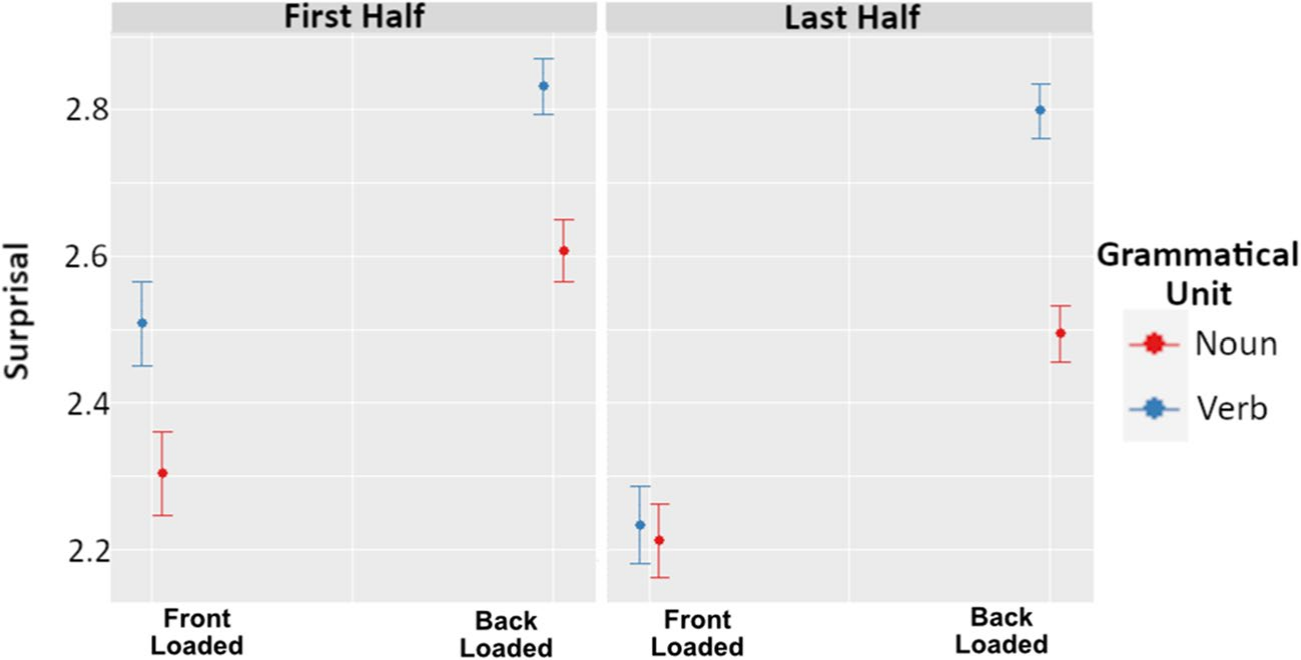
Three-way interaction between grammatical unit, structure, and utterance half on surprisal values. The left panel depicts first utterance half, and the right panel depicts last utterance half. Within each panel, values on the left side correspond to back-loaded languages, while values on the right correspond to front-loaded languages. Red values denote nouns, while blue denote verbs. The circles indicate the predicted median value, and the bands depict the 89% confidence interval. (Color figure online)

For surprisal, the best fitting model included grammatical unit, front versus back loading, and utterance half, as well as interactions between these variables, χ^2^(4)= 135.36, *p* < .001. The results of this model are visualized in Fig. 4, and specific model terms are provided in Table 2. Importantly, we see that nouns and verbs in back-loaded languages have higher surprisal (*M* = 2.745) than the same grammatical units in front-loaded languages (*M* = 2.296, *t* = 8.407), and verbs overall have higher surprisal (*M* = 2.741) than nouns (*M* = 2.457, *t* = 11.304). Finally, this noun–verb difference is greatest in the last half of utterances (*t* = 3.016), but this is primarily the case in back-loaded languages (*t* = 5.456).

**Table 2:** Model estimates for surprisal mixed-effects model

Fixed effect	Estimate	Std. Error	*t* value
Grammatical Unit (Verb > Noun)	0.225	0.019	11.314
Front Loaded > Back Loaded	−0.303	0.036	−8.407
Utterance Half (Last > First)	−0.113	0.020	−5.634
Grammatical Unit × Front Loaded > Back Loaded	−0.021	0.037	−0.056
Grammatical Unit × Utterance Half	0.079	0.026	3.016
Front Loaded > Back Loaded × Utterance Half	0.021	0.034	0.628
Grammatical Unit × Front Loaded > Back Loaded × Utterance Half	−0.263	0.048	−5.456

**Fig. 5:**
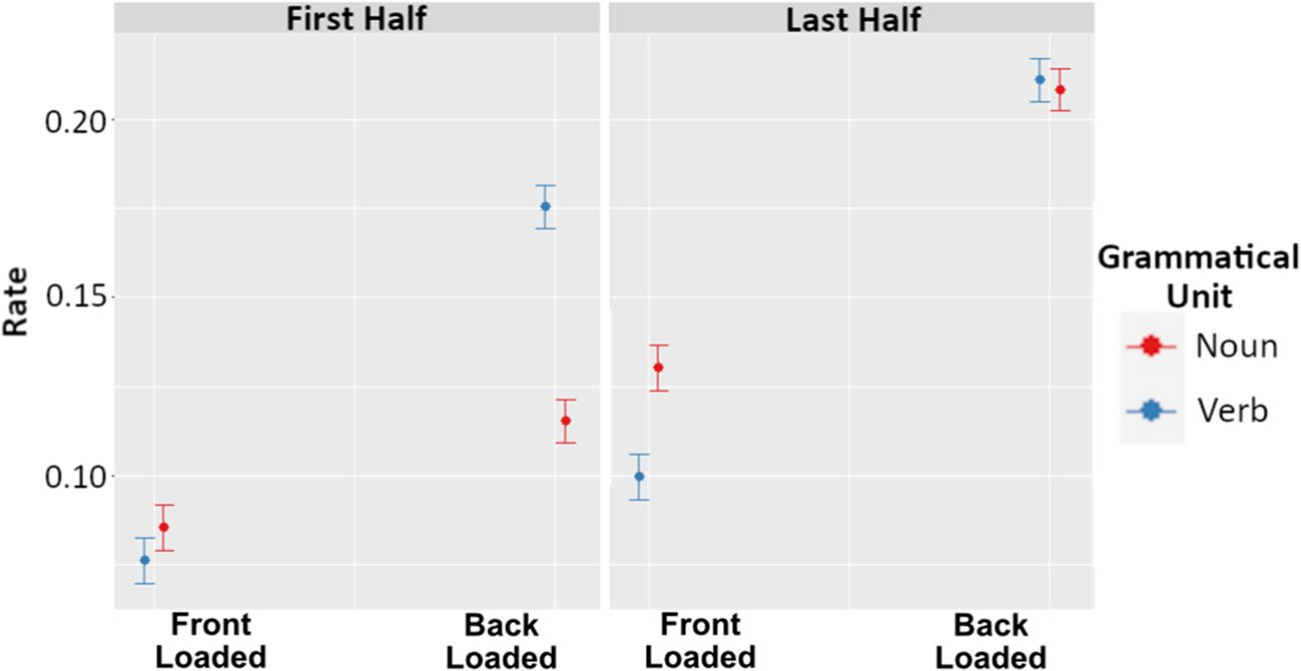
Three-way interaction between grammatical unit, front loading versus back loading, and utterance half on rate of occurrence values. The left panel depicts first utterance half, and the right panel depicts last utterance half. Within each panel, values on the left side correspond to back-loaded languages, while values on the right correspond to front-loaded languages. Red values denote nouns, while blue denote verbs. The circles indicate the predicted median value, and the bands depict the 89% confidence interval. (Color figure online)

For rate of occurrence, the best fitting model included grammatical unit, front versus back loading, and utterance half, as well as interactions between these variables, χ^2^(3) = 1038.6, *p* < .001. The results of this model are visualized in Fig. 5, and specific model terms are provided in Table 3. Importantly, we see that while front-loaded languages have generally lower rates of nouns and verbs (*M* = 0.089) compared with back-loaded languages (*M* = 0.174, *t* = 5.99), this effect is much stronger in the last half of utterances (*t* = 10.28). This indicates that back-loaded languages have a particularly high rate of nouns and verbs in the back half of the utterance.

**Table 3:** Model estimates for rate of occurrence mixed-effects model

Fixed effect	Estimate	Std. Error	t-value
Grammatical Unit (Verb > Noun)	0.265	0.008	31.98
Front Loaded > Back Loaded	−0.087	0.015	−5.99
Utterance Half (Last > First)	0.312	0.008	37.58
Grammatical Unit × Front Loaded > Back Loaded	−0.285	0.012	−24.29
Grammatical Unit × Utterance Half	−0.218	0.012	−18.59
Front Loaded > Back Loaded × Utterance Half	−0.121	0.012	−10.28
Grammatical Unit × Front Loaded > Back Loaded × Utterance Half	0.146	0.017	8.81

#### Association between noun and verb rates with surprisal differences

Next, we assessed whether such an interplay of rate, surprisal, grammatical unit, and position (i.e., utterance half) was associated with the surprisal difference (i.e., mean surprisal of last half of an utterance minus the mean surprisal of the first half). The purpose of this analysis was to build on the previous post hoc analysis in a key way. After determining that there are differences in where nouns and verbs frequently occur within an utterance across languages, and that nouns and verbs differ in their mean surprisal values as a function of their position in the utterance, the current analysis aimed to assess how our primary outcome measure for this study, surprisal difference, was related to the rates and mean surprisal values of nouns and verbs. Based on the previous analyses, we predicted that large, positive surprisal differences (i.e., back loading) would be associated with an interaction between utterance half and rate or mean surprisal. In other words, utterances that show greater back loading should show higher rates and surprisal values of nouns and verbs, primarily in the last (i.e., back) half of an utterance. If this is the case, this would be evidence that the relative distribution of nouns and verbs within an utterance (partially) explains the front- versus back-loading patterns of information distribution, even at utterance-level.

Due to the very complex nature of the model, and the potential difficulty of determining the order in which to add parameters for step-wise model building, we followed a backwards model building approach for this analysis (Barr et al., 2013). That is, we started with a full model that contained dyad/participant as random intercept, surprisal difference as the dependent variable, and rate, surprisal, utterance half, and grammatical unit as fixed factors, as well as all two-, three-, and four-way interactions. We then step-wise removed the model term with the lowest explained variance, and used the likelihood ratio test to compare this model to the model that still contained this term. If the model comparison was not significant, this indicated that the model fit was not dependent on that parameter, and we left the term out of the model. We then continued with the next least-contributing parameter, until we reached a significant comparison, indicating that removing a particular term detrimentally impacted the model fit. We then report the model comparison statistic for this model against the base model that contained only the random effects structure.

In this analysis, our final interaction model was a significantly better fit to the data than the base model: χ^2^(11) = 1455, *p* < .001. The model showed an influence of rate of occurrence, word surprisal, utterance half, and grammatical unit on surprisal difference, as anticipated by our previous analyses. An overview of the model parameters can be seen in Table 4.

**Table 4:** Model parameters of *surprisal difference* model mixed-effects model

Fixed effect	Estimate	Std. Error	t-value
Rate	0.298	0.118	2.539
Utterance Half (Last > First)	−1.345	0.064	−20.872
Word Surprisal	−0.145	0.019	−7.387
Grammatical Unit (Verb > Noun)	0.172	0.057	3.006
Rate × Surprisal	0.169	0.055	3.096
Utterance Half × Surprisal	0.236	0.025	9.412
Utterance Half × Grammatical Unit	−0.301	0.085	−3.557
Rate × Utterance Half × Surprisal	0.195	0.053	3.663
Rate × Surprisal × Grammatical Unit	−0.183	0.046	−3.938
Utterance Half × Surprisal × Grammatical Unit	0.109	0.027	4.004
Rate × Utterance Half × Surprisal × Grammatical Unit	−0.147	0.066	−2.217

#### Relationship between front loading versus back loading on turn-taking timing

Finally, to determine whether these structural (i.e., front vs. back loaded) differences are associated with the dynamics of dialogue, we also tested whether floor transfer offset (FTO; the time between the end of speaker A’s utterance and the beginning of speaker B’s utterance) differed between front-loaded and back-loaded languages, or as a function of the mean surprisal of the first or last half of the utterance.

To this end, we created a mixed model with FTO as dependent variable, number of words as a fixed effect, and dyad as random intercept (random slope led to singular fit). For the overall structure comparison, we compared this model to the same model with language structure (front vs. back loaded; treatment coded with back loaded = 0, front loaded = 1) as an additional predictor using the same likelihood ratio test described above. For the assessment of utterance mean surprisal, we including mean surprisal of the first half and last half of the utterance as predictor values.

If front- versus back-loaded information distributions are associated with turn taking dynamics, we would expect to find that back-loaded languages show higher FTOs. This would indicate that when information comes later in an utterance, the other speaker takes more time in responding.

**Fig. 6:**
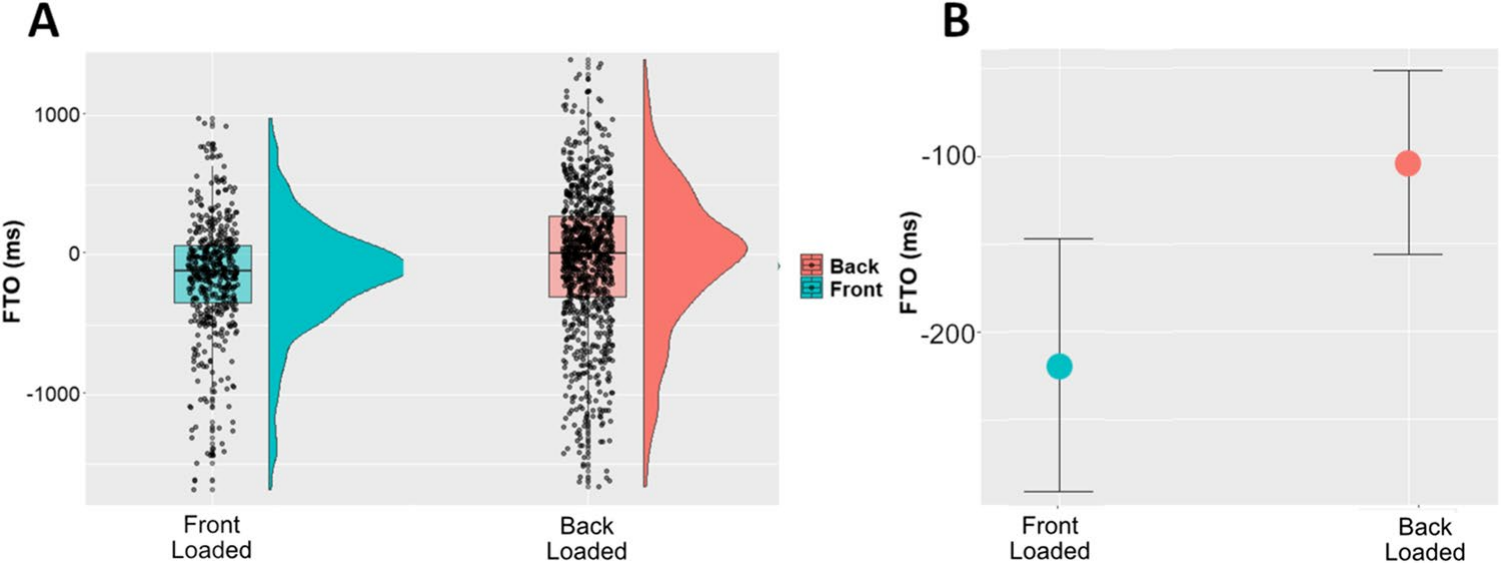
Overview of FTO differences between front-loaded and back-loaded languages. **A** Raw values, with outliers (±2 standard deviations from the mean) removed for visualization. Back-loaded languages are given on the left, in red, and front-Loaded languages are given on the right, in blue. Boxplots provide the median (notch in box), first and third quartiles (lower and upper bounds of the box), and the furthest datapoint that is maximally 1.5 times the interquartile range from the nearest bound (whiskers). The black overlaid circles depict the raw values for each utterance. The half-violins depict the data distribution densities. **B** Marginal (i.e., predicted) effects from our mixed model. Back-Loaded languages are given on the left, in red, and front-loaded languages are given on the right, in blue. In both panels, information front loading versus back loading is depicted along the *x*-axis, while FTO, in milliseconds, is given on the *y*-axis. (Color figure online)

Our test of the association between FTO and front versus back loading revealed a significant association, χ^2^(1) = 12.725, *p* < .001, with the FTO in back-loaded languages estimated to be 109.142±30.44 ms (*t* = 3.584) longer than in front-loaded languages. See Fig. 6 for an overview of these results. However, we did not find any evidence of FTO correlating with mean surprisal of either utterance half, χ^2^(2) = 0.248, *p* = .884.

## Discussion

In this study, we aimed to investigate patterns of information distribution across different languages when considering spoken dialogue. Our findings show that in conversational language use, and across all languages investigated here, information distribution differs between the first and last half of an utterance.

Specifically, our first main finding was that information density in spoken utterances differs between the first and last half of an utterance, demonstrating a generally increasing or decreasing distribution pattern. The patterns of information distribution observed here show that the distributions in utterances can be seen as back loaded or front loaded, with more high-surprisal words occurring in either the first or the second half of an utterance, depending on the language.

Our results additionally show that while individual languages show evidence of a particular dominant pattern of either front- or back-loaded information, they also group according to these patterns. Specifically, for the six languages investigated here, we found that Spanish and Mandarin showed the same back-loaded pattern as English. However, German, Japanese, and Arabic all showed front-loaded information distributions.

The information distribution within utterances that our analyses have revealed might have important implications for models of language processing, as speakers are thought to update their prediction of an utterance’s meaning while the utterance is still unfolding (McClelland et al., 1989; Rabovsky et al., 2018), and while also planning their response in parallel (Barthel & Levinson, 2020; Barthel et al., 2016; Bögels et al., 2015; Corps et al., 2018; Levinson, 2006; Levinson & Torreira, 2015; Magyari et al., 2017). This early planning can negatively affect cognitive processing of the remainder of the utterance (Barthel, 2021; Bögels et al., 2018), which the addressee must attend to while also planning their own utterance.

Our results are particularly interesting when considered together with the experimental work done by Bögels et al. (2018), who investigated the effects of planning and processing spoken language in parallel. Given that high information words occur in the latter half of utterances in some languages, an interesting route for follow-up research would be to determine whether early next-turn speech planning occurs on a similar timescale in these back-loaded languages, or whether next-turn speech planning then occurs later, after more of the on-going turn (including more of the high information words) has been uttered. The present study already provides some initial evidence for conversational dynamics differing between languages with high front-loaded information density compared with back-loaded languages. Indeed, we found that back-loaded languages have significantly longer floor transition offsets. Still, it is important to note that the effects, which are on the order of approximately 100 ms, are still within the general range of variation observed in large cross-linguistic analyses of turn transition times (Stivers et al., 2009). Additionally, there may be other factors contributing to these FTO differences that covary with the information distribution across utterances within a language, such as frequency of questions in general, or specific types of questions (e.g., tags, interrogatives, or other cultural specifics of turn-sequential organization (Kendrick et al., 2020; Kiverstein & Rietveld, 2020; Schegloff, 2007; Stivers, 2013). Future experimental work is required to control and manipulate these potential factors and test for causal relations between utterance-level information distribution and FTOs, as well as potential cognitive processing consequences and next-turn planning consequences.

At the same time, however, there is some evidence that predicting turn ends and speech acts may occur later in languages such as Japanese, despite higher information density early on. This is due to how the information incrementally builds up, and the ability to retroactively modify the grammatical structure of an utterance as it unfolds (Tanaka, 2000). To illustrate what is meant by retroactive modification, we can take an example from Tanaka (2000, pp. 32–33). After a wife has just complained about an aspect of her husband’s behavior, the husband responds first with *sore wa soo ne* [that’s right, isn’t it”], a syntactically complete utterance indicating agreement. After a pause, he continues with *tto iu kara ikenai no* [“(it’s) wrong because (I) say that”]. This addition changes the first utterance into a quotation with the word *tto,* which transforms the stance of the entire utterance. The two utterances create one well-formed syntactic unit: *sore wa soo ne tto iu kara ikenai no* [(It’s) wrong because (I) say that “that’s right, isn’t it”]. The speaker’s stance in *sore wa soo ne* changes from agreement into self-deprecation in the course of one compound utterance (Tanaka, 2000). The late prediction that results from such linguistic structure may be offset by the relatively lower information density in the latter half of the utterance that we found in the present study. While Japanese has a very high degree of incrementality, which may be relevant for how information distribution impacts processing for the addressee, German has also been reported to show a greater degree of incrementality when compared with English, although less so than Japanese (Couper-Kuhlen & Ono, 2007). Testing these ideas, and specifically how information distribution (as measured by word surprisal), incrementality and word order interact in incurring processing costs as a speaking turn unfolds, would require a cross-linguistic comparison of comprehension effort, and should be addressed with future research.

Additionally, our post-hoc analyses aimed to determine if higher first- or last-half surprisal was related to the frequency of occurrence of nouns and verbs across the two utterance halves, and the magnitude of surprisal of these particular words. In other words, we predicted that the higher surprisal utterance half would have a higher rate of nouns and verbs, and potentially that nouns and verbs in the higher surprisal utterance half would also have higher mean surprisal values than nouns and verbs in the lower surprisal half. We found that the rate of nouns and verbs differed according to both the utterance half, and whether the language is front-loaded or back-loaded. Overall, we see that back-loaded languages have particularly high rates of occurrence of nouns and verbs in the last (back) half of utterances. Additionally, we see that verbs, which have higher mean surprisal values than nouns in back-loaded languages, also have particularly high surprisal values in the last half of utterances. Together, these additional analyses provide some evidence that the higher-surprisal half of an utterance is characterized by a greater frequency of highly informative grammatical units (i.e., nouns and verbs), and that these words have particularly high surprisal values when found in the more information-heavy half of the utterance. These findings are in line with other research that has argued for a link between the placement of particular high-informative grammatical units and information distribution more broadly (Hahn & Xu, 2022; Maurits, 2012; Roberts & Levinson, 2017). Our results therefore suggest that the way a language orders particular grammatical units within an utterance influences the surprisal value of these words, and this interaction may be contributing to the higher-level information distribution pattern observed at the language level.

Our findings also provide more evidence that theories of communication and language organization cannot be based on English alone. In particular, there does not seem to be any one-to-one mapping of the “classic” word order structures such as subject-verb-object (SVO) versus verb-subject-object (VSO) or subject-object-verb (SOV) and the front/back loading of information revealed by the current study. Our second post hoc analysis suggests a relationship between the rate of occurrence of particular grammatical units and the actual surprisal value of these units, and show that a complex interplay between the rate of nouns and verbs, the specific surprisal values that they carry, and whether they occur in the first or last half of an utterance is associated with the (im)balance of surprisal values between the first and last half of the utterance. These results suggest that such an interplay of linguistic structure, at the level of the ordering of particular grammatical units across an utterance, relates to how much information these words carry. These structural dynamics may then result in the particular information distribution patterns that we observed, which will differ across different languages. However, it is important to note that these effects are likely further embedded in the larger ecology of interaction. For example, word order in natural utterances may depend on other factors, such as tense or speech act. Future studies should therefore also assess whether and how the information distribution as presented in the present study changes according to more fine-grained linguistic and conversational structure. For example, prosodic cues, speech rhythm, and co-articulation, as well as the frequency of particular question structures, could all contribute to the information distribution patterns, as well as the impact such patterns have on conversation dynamics.

Based on Klafka and Yurovsky’s (2021) analysis of language families, it is also possible that these findings can be predictive of how other languages within the same phylogenetic family may behave. Understanding these information distribution patterns on the scale of language families will, however, require future research to compare a larger set of diverse languages, using well-matched corpora of spoken language use. Additionally, these cross-linguistic differences could be informative for understanding and investigating broader phenomena in human communication, such as multimodal patterns of communicative behavior in face-to-face dialogue, as well as the dynamics of dyadic information exchange, and how these patterns and dynamics may function differently depending on the language.

## Limitations

While we interpret our results as suggesting that languages either have generally heavier information density in the first half or the last half of an utterance, it is also possible that other patterns lead to these same overall findings. For example, there may be an increase in surprisal in the middle of the utterance that is slightly higher in the first or second half of the utterance. While our analysis methods do not allow us to detect such complex patterns, large-scale studies of information distribution (e.g., Klafka & Yurovsky, 2021) suggest that information density is either smoothly increasing, or smoothly decreasing, rather than having a higher-order shape. However, future studies should assess whether such high-order polynomials may better explain the shape of information curves in different languages. Additionally, very little is known about the extent to which early planning occurs, and impacts parallel comprehension, beyond the studies in the Dutch language. Therefore, it is important for future work to experimentally assess parallel planning and comprehension in other languages, and to assess to what extent it is impacted by the information distribution of the language, and of the individual utterances being tested. Finally, it should be noted that, in order to ensure that the utterances analyzed contained a sufficient number of words for our surprisal calculations, we had to systematically exclude both the first two words of each utterance, and many shorter utterances, meaning that our final analyses are based on a subset of all utterances occurring in the corpus. Regarding the exclusion of the first two words, it may be that these words contribute to the overall distribution of information in ways that cannot be quantified using the surprisal calculations used in the current study. Therefore, alternative, complementary measures of information distribution should be employed to quantify and compare distributions of shorter utterances. Related to this, future work could also investigate whether shorter utterances, which occur frequently in conversation, show a different pattern of information distribution.

## Conclusion

In sum, this study shows that in the spoken modality, information distribution follows either an increasing or decreasing pattern. While individual languages seem to show a particular pattern, there is not one dominant pattern across even the six languages investigated in this study. These patterns of information distribution may further impact the broader organization of multimodal communicative behavior and the dynamics of conversation.

## Data Availability

All associated data and analysis files can be found on the Open Science Framework project site: https://osf.io/naw4m/.
